# An observational study of adults seeking emergency care in Cambodia

**DOI:** 10.2471/BLT.14.143917

**Published:** 2014-12-08

**Authors:** Lily D Yan, Swaminatha V Mahadevan, Mackensie Yore, Elizabeth A Pirrotta, Joan Woods, Koy Somontha, Yim Sovannra, Maya Raman, Erika Cornell, Christophe Grundmann, Matthew C Strehlow

**Affiliations:** a Department of Surgery, Stanford University School of Medicine, 300 Pasteur Drive, Stanford, CA 94305, United States of America (USA).; b University Research Co. Centre for Human Services, Phnom Penh, Cambodia.; c Deutsche Gesellschaft für Internationale Zusammenarbeit, Phnom Penh, Cambodia.; d Feinberg School of Medicine, Northwestern University, Chicago, USA.

## Abstract

**Objective:**

To describe the characteristics and chief complaints of adults seeking emergency care at two Cambodian provincial referral hospitals.

**Methods:**

Adults aged 18 years or older who presented without an appointment at two public referral hospitals were enrolled in an observational study. Clinical and demographic data were collected and factors associated with hospital admission were identified. Patients were followed up 48 hours and 14 days after presentation.

**Findings:**

In total, 1295 hospital presentations were documented. We were able to follow up 85% (1098) of patients at 48 hours and 77% (993) at 14 days. The patients’ mean age was 42 years and 64% (823) were females. Most arrived by motorbike (722) or taxi or tuk-tuk (312). Most common chief complaints were abdominal pain (36%; 468), respiratory problems (15%; 196) and headache (13%; 174). Of the 1050 patients with recorded vital signs, 280 had abnormal values, excluding temperature, on arrival. Performed diagnostic tests were recorded for 539 patients: 1.2% (15) of patients had electrocardiography and 14% (175) had diagnostic imaging. Subsequently, 783 (60%) patients were admitted and 166 of these underwent surgery. Significant predictors of admission included symptom onset within 3 days before presentation, abnormal vital signs and fever. By 14-day follow-up, 3.9% (39/993) of patients had died and 19% (192/993) remained functionally impaired.

**Conclusion:**

In emergency admissions in two public hospitals in Cambodia, there is high admission-to-death ratio and limited application of diagnostic techniques. We identified ways to improve procedures, including better documentation of vital signs and increased use of diagnostic techniques.

## Résumé

**Objectif:**

Décrire les caractéristiques et les principales affections des adultes qui ont recours aux soins d'urgence dans deux hôpitaux cambodgiens provinciaux de référence.

**Méthodes:**

Les adultes âgés de plus de 18 ans qui se sont présentés sans rendez-vous à deux hôpitaux publics de référence, ont été inscrits à une étude observationnelle. Les données cliniques et démographiques ont été recueillies et les facteurs associés à l'hospitalisation ont été identifiés. Les patients ont été suivis 48 heures et 14 jours après leur présentation.

**Résultats:**

Au total, 1295 présentations à l'hôpital ont été documentées. Nous avons pu suivre 85% (1098) des patients 48 heures et 77% (993) 14 jours après leur présentation. L'âge moyen des patients était de 42 ans et 64% (823) des patients étaient des femmes. La plupart des patients étaient arrivés en moto (722), en taxi ou en tuk-tuk (312). Les principales affections communes étaient en majorité des douleurs abdominales (36%; 468), des problèmes respiratoires (15%; 196) et des maux de tête (13%; 174). Parmi les 1050 patients dont les signes vitaux ont été enregistrés, 280 avaient des valeurs anormales, hormis la température, lors de leur arrivée. Les tests diagnostiques réalisés ont été enregistrés pour 539 patients: 1,2% (15) des patients avait bénéficié d'une électrocardiographie et 14% (175) des patients avaient bénéficié de l'imagerie diagnostique. Par la suite, 783 (60%) patients ont été hospitalisés et 166 d'entre eux ont subi une opération chirurgicale. Les indicateurs significatifs d'hospitalisation comprenaient l'apparition des symptômes dans les 3 jours précédant la présentation, des signes vitaux anormaux et la fièvre. À l'issue du suivi des 14 jours, 3,9% (39/993) des patients étaient décédés et 19% (192/993) des patients présentaient des capacités fonctionnelles diminuées.

**Conclusion:**

Dans les hospitalisations d'urgence dans les deux hôpitaux publics au Cambodge, il y a un rapport hospitalisation-décès élevé et une application limitée des techniques de diagnostic. Nous avons identifié des moyens pour améliorer les procédures, y compris la meilleure documentation des signes vitaux et l'utilisation accrue des techniques de diagnostic.

## Resumen

**Objetivo:**

Describir las características y quejas principales de los adultos que solicitan atención de emergencia en dos hospitales provinciales de remisión de Camboya.

**Métodos:**

Se inscribió a adultos mayores de 18 años que acudieron sin cita previa a dos hospitales públicos de remisión en un estudio observacional. Se recogieron datos clínicos y demográficos y se identificaron los factores relacionados con el ingreso hospitalario. Se realizó un seguimiento a los pacientes durante 48 horas y 14 días después de la visita hospitalaria.

**Resultados:**

En total, se documentaron 1295 visitas hospitalarias. Se logró realizar un seguimiento del 85 % (1098) y del 77 % (993) de los pacientes a las 48 horas y a los 14 días, respectivamente. La edad media de los pacientes fue de 42 años y el 64 % (823) eran mujeres. La mayoría acudió en motocicleta (722), taxi o tuk-tuk (312). Las quejas principales más comunes fueron dolor abdominal (36 %; 468), problemas respiratorios (15 %; 196) y cefalea (13 %; 174). De los 1050 pacientes con registro de constantes vitales, 280 presentaron valores anómalos, a excepción de la temperatura corporal, a su llegada. Se registraron las pruebas diagnósticas realizadas en 539 pacientes: se realizó una electrocardiografía al 1,2 % (15) e imágenes diagnósticas al 14 % (175) de los pacientes. Posteriormente, 783 (60 %) de los pacientes fueron ingresados, de los cuales 166 se sometieron a cirugía. Los predictores significativos para el ingreso incluyeron la aparición de los síntomas 3 días antes de la visita hospitalaria, constantes vitales anómalas y fiebre. Antes de finalizar el seguimiento de 14 días, el 3,9 % (39/993) de los pacientes había fallecido y el 19 % (192/993) seguía presentando discapacidades funcionales.

**Conclusión:**

Las hospitalizaciones de emergencia de dos hospitales públicos de Camboya presentan una elevada relación entre ingreso y defunción, así como un uso limitado de las técnicas de diagnóstico. Se identificaron formas de mejorar los procedimientos, como una mejor documentación de las constantes vitales y un mayor uso de las técnicas de diagnóstico.

## ملخص

**الغرض:**

وصف خصائص البالغين الذين يلتمسون رعاية الطوارئ في مستشفيين للإحالة على مستوى المقاطعات في كمبوديا وشكاواهم الرئيسية.

**الطريقة:**

تم تسجيل البالغين في سن 18 عاماً فأكثر الذين تواجدوا في مستشفيين عامين للإحالة دون موعد في دراسة قائمة على الملاحظة. وتم جمع البيانات السريرية والديمغرافية وتحديد العوامل ذات الصلة بدخول المستشفى. وتم متابعة المرضى لمدة 48 ساعة و14 يوماً بعد دخولهم إلى المستشفى.

**النتائج:**

بشكل إجمالي، تم توثيق 1295 حالة دخول إلى المستشفى. وتمكنا من متابعة 85 % (1098) من المرضى لمدة 48 ساعة و77 % (993) لمدة 14 يوماً. وكان متوسط عمر المرضى 42 سنة وكانت نسبة الإناث 64 % (823). وكان وصول معظمهم إلى المستشفى باستخدام الدراجة النارية (722) أو سيارة الأجرة أو التوك توك (312). وكانت أكثر الشكاوى الرئيسية شيوعاً ألم بالبطن (36 %؛ 468 شخصاً) ومشكلات تنفسية (15 %؛ 196 شخصاً) والصداع (13%؛ 174 شخصاً). ومن أصل 1050 مريضاً تم تسجيل علاماتهم الحيوية لدى وصولهم، كان لدى 280 مريضاً قيم شاذة، باستثناء درجة الحرارة. وتم على النحو التالي تسجيل الاختبارات التشخيصية التي تم إجراؤها بخصوص 539 مريضاً: خضع 1.2 % (15) من المرضى لإجراء تخطيط كهربائية القلب وخضع 14 % (175) لإجراء التصوير التشخيصي. وتلى ذلك إدخال 783 (60 %) مريضاً إلى المستشفى وخضع 166 مريضاً من هؤلاء المرضى إلى الجراحة. واشتملت عوامل التكهن الهامة بدخول المستشفى على بدء الأعراض قبل دخول المستشفى بثلاثة أيام وشذوذ العلامات الحيوية والحمى. وأسفرت نتائج المتابعة التي بلغت مدتها 14 يوماً عن وفاة 3.9 % (39/993) وظل 19 % (192/993) يعانون من قصور وظيفي.

**الاستنتاج:**

ثمة ارتفاع في نسبة دخول المستشفى إلى الوفاة ومحدودية في استخدام تقنيات التشخيص لدى دخول المستشفى في حالات الطوارئ في مستشفيين عامين في كمبوديا. وقمنا بتحديد طرق لتحسين الإجراءات، بما في ذلك تحسين توثيق العلامات الحيوية وزيادة استخدام تقنيات التشخيص.

## 摘要

**目的:**

描述两所柬埔寨省级转诊医院成年人寻求急诊治疗的特征和主诉。

**方法:**

将两所公共转诊医院没有预约的18岁及以上成年人纳入观察性研究。收集临床和人口数据，并确定与入院相关的因素。在就医48小时和14天后对患者进行跟踪随访。

**结果:**

总计记录了1295个医院就医情况。我们能够在48小时随访85%（1098）的病人，在14天随访77%（993）的病人。病人平均年龄为42岁，64%（823）为女性。大多数通过摩托车（722）或者的士或嘟嘟车（312）前来就医。最普遍的主诉是腹痛（36%；468）、呼吸道问题（15%；196）和头痛（13%；174）。在记录生命体征的1050位患者中，280位抵达时有异常值（除体温外）。对539位病人记录了执行过的诊断性测试：1.2%（15）病人有心电图，14%（175）有诊断性影像。其后，783（60%）位病人入院，其中166位病人接受了手术。住院的显著预测因子包括前来就医之前3天内的症状发作、异常生命体征和发烧。截至14天随访时，有3.9%（39/993）病人死亡，19%（192/993）病人依然保持功能性受损状态。

**结论:**

在柬埔寨两所公立医院的紧急住院中，住院死亡率较高，诊断技术的应用有限。我们确定改进流程的方法，包括更好的生命体征病案记录和对诊断技术的更多使用。

## Резюме

**Цель:**

Описать характеристики и основные жалобы взрослых, обратившихся за неотложной помощью в два камбоджийских провинциальных лечебно-диагностических центра.

**Методы:**

В данное обсервационное исследование были включены взрослые старше 18 лет, которые самостоятельно, без назначения, обратились в два государственных лечебно-диагностических центра. Были собраны клинические и демографические данные и определены факторы, связанные с госпитализацией. Пациенты повторно наблюдались спустя 48 часов и 14 дней после обращения в центры.

**Результаты:**

В общей сложности, было задокументировано 1295 случаев обращения в центры. Наблюдение велось за 85% (1098) пациентов спустя 48 часов и 77% (993) пациентов спустя 14 дней. Средний возраст пациентов составлял 42 года, и 64% (823) из них были женщины. Большинство пациентов прибыли в центры на мотоцикле (722), на такси или тук-туке (312). Основными, наиболее распространенными жалобами являлись боли в области живота (36%; 468), нарушения со стороны дыхательной системы (15%; 196) и головная боль (13%; 174). Из 1050 пациентов с зарегистрированными основными показателями жизнедеятельности у 280 пациентов по прибытии были выявлены несоответствующие нормам показатели жизнедеятельности, за исключением температуры. Результаты диагностических исследований были зарегистрированы у 539 пациентов: 1,2% (15) пациентов прошли электрокардиографию и 14% (175) пациентов – диагностическую визуализацию. Впоследствии 783 (60%) пациента были госпитализированы и 166 из них перенесли операцию. В число значимых параметров для госпитализации входили следующие: появление симптомов в течение трех дней до обращения, несоответствующие нормам показатели жизнедеятельности и повышенная температура. При проведении 14-дневного наблюдения выяснилось, что 3,9% (39/993) пациентов скончались и 19% (192/993) оставались с ограниченной подвижностью.

**Вывод:**

При проведении экстренной госпитализации в двух государственных больницах в Камбодже наблюдается высокий коэффициент смертности относительно числа принимаемых на лечение пациентов и ограниченное применение диагностических методов. Были определены способы улучшения процедур лечения, включая улучшенное документирование основных показателей жизнедеятельности и более частое использование диагностических методов.

## Introduction

Emergency medicine is a neglected component of health-care systems in most low- and middle-income countries. Nearly half of deaths and one third of disability adjusted life-years lost in these countries could be avoided by applying the basic principles of emergency care.^1^ However, despite the renewed interest shown by World Health Organization calls to expand emergency care infrastructure^2^ – these systems remain underdeveloped in most low- and middle-income countries and access to high-quality care is limited.^1^^,^^3^^,^^4^

The current health-care system in Cambodia exemplifies this gap in the provision of essential emergency care. Since the Cambodian Ministry of Health was established in 1993, the country has relied on a combination of public and private providers and international nongovernmental organizations to fulfil the medical needs of its people. In 2012, in the public sector, 1080 health centres provided primary care, first aid and maternal and child care (designated the minimum package of activity) in rural areas, while 90 referral hospitals provided secondary and tertiary care – designated the complementary package of activity. Hospitals offering care at complementary package of activity level 2 and above are required to provide emergency care at all times and each has an onsite physician and nurse, with a midwife available for obstetric emergencies.^5^ Nevertheless, many of these hospitals lack designated emergency departments, a formal triage process and staff trained in emergency medicine. Furthermore, emergency medicine is not recognized as a specialty by the Ministry of Health and there is no formal programme for training residents in the discipline.

As functional emergency care systems vary between countries and regions,^3^ accurate characterization of patients seeking emergency care is essential for developing locally appropriate systems. The 2013 academic emergency medicine consensus conference identified the lack of data on chief complaints as a critical gap in global emergency care research.^6^ The chief complaint is the primary consideration used by health-care providers to structure the evaluation and management of patients who present with an acute condition or with an acute presentation of a chronic disease. Moreover, the information obtained from a good understanding of chief complaints differs from that gleaned from discharge diagnoses.^7^ In countries with mature emergency care systems, chief complaints are standardized and mapped.^8^ However, few developing nations have the means to report or collate information on chief complaints,^6^ even though these data are essential for resource allocation, training, research and syndromic surveillance.

The objectives of this study were to identify the characteristics and chief complaints of adults presenting for emergency care in Cambodia. Understanding the pattern of adult emergencies in Cambodia could help guide the development of policy on health-care interventions, such as triage systems and staff training for common conditions. This information could also provide a basis for future studies of the effectiveness and quality of emergency medical services and of patient outcomes.

## Methods

We conducted a four-week observational study of unscheduled visits to two provincial government referral hospitals in Cambodia: Sampov Meas Provincial Hospital and Battambang Provincial Hospital. Both were classified at complementary package of activity level 3, which indicates they provided obstetric, emergency and surgical services in addition to a variety of specialty services. Sampov Meas Provincial Hospital has 162 inpatient beds and reported a total of 16 426 visits in 2012, which resulted in 6704 admissions. Battambang Provincial Hospital is a larger facility with 220 inpatient beds. It reported 55 138 visits in 2012, resulting in 14 411 admissions. All figures come from the Cambodian Ministry of Health database.^9^ At the start of the study, neither of the hospitals had a designated department for admissions, and Battambang Provincial Hospital did not have an emergency department. In effect, patients triaged themselves and initially presented to various locations, such as the emergency department, the intensive care unit, different wards and the operating room. Consequently, the study enrolled patients who presented throughout the hospital, not only at the emergency department. The study was approved by institutional review boards at Stanford University School of Medicine in the United States of America and the Cambodian Ministry of Health.

We enrolled all patients aged 18 years or older who presented at the two study hospitals without an appointment between each Sunday and Friday evening during four consecutive weeks in July and August 2012. Because of study logistics, data collection at weekends differed between the two hospitals. At Sampov Meas Provincial Hospital, patients who presented between 17:00 on Friday and 17:00 on Sunday and who were still in hospital on Monday morning were enrolled; at Battambang Provincial Hospital, no weekend patients were enrolled. A repeat unscheduled visit was considered a separate visit. Verbal consent was obtained from patients or guardians and interviews were conducted in Khmer through native-speaking translators.

Patients presented infrequently during weekends when hospital staffing was limited. Sensitivity analyses showed that including the 63 weekend patients we enrolled did not substantially alter our study results: inclusion resulted in odds ratios (ORs) for predictors varying by around 2%. Thus, weekend patients were included in our multivariate analysis of surgery and functional impairment but not of admissions.

Demographic and clinical data were collected by one research team at each hospital using a standardized, secure, web-based application: Research Electronic Data Capture (REDCap, Vanderbilt University, Nashville, USA), which is designed to support data capture for research studies.^10^ Up to three chief complaints were recorded for each patient. The full list of chief complaints is available in Appendix A (available from: http://emed.stanford.edu/education/international/pubs.html). Information was collected from patients, their friends or family and hospital staff and records, and was entered into REDCap via tablet computers connected to mobile telecommunications networks. Two research staff reviewed each patient record for consistency and inconsistencies were resolved by repeating patient interviews and by reviewing hospital records. Follow-up data on patient outcomes were collected 48 hours and 14 days after the patient’s initial visit, either directly from patients remaining in hospital or by telephone. Patients were considered lost to follow-up if they could not be contacted on three successive days.

### Data analysis

The study’s primary outcomes were admission, medical interventions, surgery, functional impairment and death. In Cambodia, patients may remain in the emergency department for treatment and monitoring for a week or more without being transferred to another department. We recorded only interventions completed within 48 hours of the initial presentation, thereby identifying the most urgent. Patients who stayed in hospital overnight were regarded as having been admitted. Functional impairment was defined as significant pain, significant limitation in performing daily activities, confinement to bed or a comatose state.

Multivariate modelling was performed to identify factors associated with admission, surgery and functional impairment. To avoid including patients who underwent minor procedures and were treated and released, we only used data on admitted patients when identifying factors were associated with surgery. Although we included women who presented with presumed labour in the demographic data, we excluded them from our analysis of outcomes in admitted patients and from multivariate analyses since all women in labour were admitted. As vital signs were commonly not observed or documented, we included records with missing data for the respiratory rate, blood pressure or heart rate in the multivariate analyses by assuming that absent values were normal. Records with missing values for any other independent variable were excluded.

Multivariate logistic regression models, which controlled for age and gender, were built for the primary outcomes using predictors identified by univariate analysis. Stepwise methods were not used. Statistical analysis was performed using SAS Enterprise Guide for Windows, version 4.3 (SAS Institute Inc., Cary, USA). ORs and 95% confidence intervals (CI) were calculated for all model variables.

## Results

During the study period, 2162 visits without a prior appointment were documented at the two study hospitals. Of these, 1295 (59.9%) were visits by adult patients aged 18 years or older; these patients comprised the study cohort. We were able to follow up 84.8% (1098) of patients at 48 hours and 76.7% (993) at 14 days. The patients’ mean age was 42 years and most patients were female and employed (Table 1). The predominant mode of transport to hospital was by private vehicle: 55.8% (722) of patients arrived by motorbike and 24.1% (312), by taxi or tuk-tuk, whereas only 9.3% (120) arrived by ambulance. Travel times to the study hospitals were typically short (i.e. less than 2 hours).

**Table 1:** Adults presenting without appointments at two Cambodian hospitals, July–August, 2012

Characteristic	No. (%) of patients^a,b^
**All patients**	1295 (100)
**Demographic characteristic**	
Site of presentation	
Battambang Provincial Hospital	678 (52.4)
Sampov Meas Provincial Hospital	617 (47.6)
Age	
Age in years, mean (SD)	42.0 (17.1)
Aged 18–64 years	1132 (87.4)
Aged 65 years or more	163 (12.6)
Sex	
Female	823 (63.6)
Male	472 (36.4)
**Socioeconomic characteristic**	
Patient had low-income health insurance^c^	572 (44.2)
Occupation	
Employed	809 (62.5)
Unemployed	380 (29.3)
Other, such as a student or monk	70 (5.4)
**Travel to hospital**	
Time	
Time in hours, median (IQR)	0.5 (0.3–1.3)
Time < 0.5 hours	670 (51.7)
Time 0.5–2 hours	495 (38.2)
Time > 2 hours	98 (7.6)
Distance	
Distance in kilometres, median (IQR)	17 (5–32)
Distance ≤ 30 km	930 (71.8)
Distance 31–60 km	179 (13.8)
Distance > 60 km	139 (10.7)
**Presentation**	
Time of arrival	
Monday to Friday 07.00–17.00	1069 (82.5)
Overnight Sunday to Friday (i.e. 17.00–07.00)	163 (12.6)
Weekend (i.e. Friday 17.00 to Sunday 17.00)	63 (4.9)
Care before presentation	
Transferred from another health-care facility	316 (24.4)
Referred by an external medical provider	170 (13.1)
Prior care of those not transferred or referred	
Prior care received in the previous 48 hours	25 (1.9)
Prior care received more than 48 hours earlier	106 (8.2)
No prior care	627 (48.4)
**Examination findings**	
Respiratory rate, blood pressure and heart rate	
Abnormal respiratory rate, blood pressure or heart rate	280 (21.6)
Abnormal respiratory rate	88 (6.8)
Abnormal blood pressure	103 (8.0)
Abnormal heart rate	181 (14.0)
Respiratory rate, blood pressure or heart rate not measured	245 (18.9)
Temperature	
Low temperature (< 36 °C)	99 (7.6)
High temperature (> 38 °C)	97 (7.5)
Temperature not measured	285 (22.0)
Pain	
Pain present	916 (70.7)
No pain	284 (21.9)
**Risky behaviour in the previous 24 hours**	
No risky behaviour	1092 (84.3)
Tobacco use	98 (7.6)
Alcohol use	88 (6.8)
Illegal drug use	2 (0.2)

IQR: interquartile range; SD: standard deviation.

^a^ Number of patients and percentages, unless otherwise stated.

^b^ Not all categories sum to 100% as some values are missing.

^c^ Most of the other patients paid in cash, except for the more affluent who had health insurance.

Overall, 31.4% (407) of patients presented with an acute complaint (i.e. onset within the past 24 hours), 20.4% (264) with a recent complaint (i.e. symptom onset within 1 to 3 days) and 24.4% (316) with a subacute complaint (i.e. within 4 to 14 days). More than one third were transferred directly to the study hospital from another health-care facility or referred by a medical provider; only 10.1% (131) had received prior care for their chief complaint and had not been transferred from another facility or referred by a medical provider (Table 1).

**Fig. 1:**
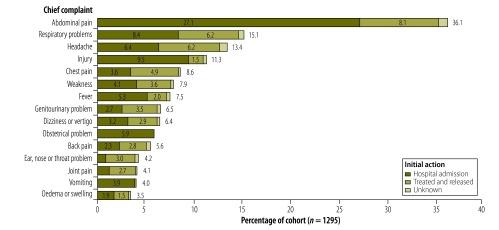
Chief complaints^a,b^ and initial actions for adults presenting without appointments at two Cambodian hospitals, July–August, 2012

^a^ Up to three chief complaints were recorded per patient.

^b^ Only the 15 most common chief complaints are shown.

Abdominal pain was the chief complaint of 36.1% (468) of patients. Fifteen percent (196) of patients reported respiratory problems and 13.4% (174) reported headache. Only 4.0% (52) of patients presented with vomiting and 1.8% (23) with diarrhoea. Fever was a chief complaint in only 7.5% (97) of cases. Injuries accounted for 11.3% (146) of visits and one death. The proportion of patients admitted was higher for those complaining of abdominal pain, injury, obstetrical problems or vomiting (Fig. 1).

The overall admission rate was 60.5% (783) and the median hospital stay was 3.9 days (Table 2). Of those admitted, 166 patients underwent surgery. Obstetric surgery accounted for 39.1% (65/166) of all surgical procedures (Table 2). Abdominal pain accounted for 44.8% (351) of all admissions. Even after women with abdominal pain associated with presumed labour were excluded from the analysis, abdominal pain still accounted for 32.3% (253) of all admissions.

**Table 2:** Actions, surgical interventions and follow-up in adults presenting without appointments at two Cambodian hospitals, July–August, 2012

Outcome	No. (%) presenting^a ^(*n* = 1295)^b^
**Initial action**	
Patient treated and released	468 (36.1)
Patient transferred to another centre^c^	11 (0.9)
Patient left hospital without being seen or against medical advice	20 (1.5)
Patient died within 24 hours of presentation	6 (0.5)
Patient admitted	783 (60.5)
Surgery	
Patient had any surgical procedure^d^	166 (15.1)
Patient had obstetric surgery^d^	65 (5.9)
Length of stay for patients admitted in days, mean (SD)	3.9 (4.8)
Cumulative mortality at 14 days^e^	39 (3.9)
**48-hour follow-up**	
Patient followed up at 48 hours	1098 (84.8)
Patient remained functionally impaired^d,f^	653 (59.5)
Patient seen by another health-care provider after discharge^d^	44 (4.0)
Patient returned to work^g^	198 (27.0)
Patient died between 24 and 48 hours^d^	11 (1.0)
**14-day follow-up**	
Patient followed up for 14 days	993 (76.7)
Patient remained functionally impaired^e,f^	192 (19.3)
Patient seen by another health-care provider after discharge^e^	122 (12.3)
Patient returned to work^h^	362 (51.5)
Patient died between 48 hours and 14 days^e^	22 (2.2)

SD: standard deviation.

^a^ Number presenting and percentage, unless otherwise stated.

^b^ Percentage of 1295 patients presenting, unless otherwise indicated.

^c^ Includes only patients transferred to another centre without first being admitted and receiving care. An additional five patients were transferred after being admitted.

^d^ Percentage of the 1098 patients followed up for 48 hours.

^e^ Percentage of the 993 patients followed up for 14 days.

^f^ Functional impairment was defined as significant pain, significant limitation in performing daily activities, confinement to bed or a comatose state.

^g^ Percentage of the 732 patients followed up for 48 hours who worked before visiting hospital.

^h^ Percentage of the 703 patients followed up for 14 days who worked before visiting hospital.

Vital signs were either not observed or not recorded for around 20% (245) of patients (Table 1) and 58.4% (756) did not receive any diagnostic tests. The investigations most frequently ordered within 48 hours were laboratory tests and diagnostic imaging (Table 3). Other investigations, such as electrocardiography, were rarely done (1.2% [15]). Computed tomography and magnetic resonance imaging were unavailable at the two hospitals. In the first 48 hours, the intervention most commonly received was medication; (84.1% [1089] of all patients), 40.5% (524) received intravenous fluids (Table 3).

**Table 3:** Diagnostic tests and interventions within 48 hours in adults presenting without appointments at two Cambodian hospitals, July–August 2012

Diagnostic test or intervention	No. (%) presenting^a,b ^(*n* = 1295)	No. (%) admitted^a,c ^(*n* = 783)
**Diagnostic test**		
Any test	539 (41.6)	464 (59.3)
Laboratory test	457 (35.3)	415 (53.0)
Diagnostic imaging	175 (13.5)	141 (18.0)
Electrocardiography	15 (1.2)	14 (1.8)
Ultrasound scanning	6 (0.5)	6 (0.8)
Diagnostic peritoneal lavage	4 (0.3)	4 (0.5)
**Medication administered**		
Any medication	1089 (84.1)	666 (85.1)
Analgesic (excluding aspirin)	688 (53.1)	460 (58.7)
Antibiotic	471 (36.4)	325 (41.5)
Antiparasitic	169 (13.1)	107 (13.7)
Aspirin	43 (3.3)	2 (0.3)
Drug administered by nebulizer	13 (1.0)	9 (1.1)
Antimalarial	9 (0.7)	9 (1.1)
Antituberculosis drug	2 (0.2)	2 (0.3)
Anti-HIV drug	2 (0.2)	1 (0.1)
Other	772 (59.6)	475 (60.7)
**Other interventions**		
Any intervention other than medication	627 (48.4)	598 (76.4)
Intravenous fluids	524 (40.5)	513 (65.5)
Wound closure	128 (9.9)	125 (16.0)
Intravenous medication	127 (9.8)	124 (15.8)
Emergency childbirth	118 (9.1)	118 (15.1)
Urethral catheterization	93 (7.2)	89 (11.4)
Oxygen therapy	81 (6.3)	76 (9.7)
Emergency cooling	49 (3.8)	49 (6.3)
Other	187 (14.4)	171 (21.8)

HIV: human immunodeficiency virus.

^a^ Categories may sum to more than 100% because some patients had more than one diagnostic test or intervention.

^b^ Overall, 42 (3.2%) patients did not undergo any diagnostic test or receive any intervention and, for 76 (5.9%) patients, no information on diagnostic tests or interventions was collected.

^c^ Overall, 8 (1.0%) admitted patients did not undergo any diagnostic test or receive any intervention and, for 49 (6.3%) admitted patients, no information on diagnostic tests or interventions was collected.

Overall, 14-day mortality in our cohort was 3.9% (39/993). Nearly half of the deaths occurred in the first 48 hours (Table 2). No patient who was treated and discharged or who underwent surgery died during follow-up. However, 34 patients with conditions that hospital staff deemed untreatable were discharged. By 14 days, six of these patients had died, five were still alive and 23 were lost to follow up. There were 232 pregnancy-related visits and of them 205 pregnant patients were past 20 weeks’ gestation. Of the pregnant women past 20 weeks’ gestation, 81.9% (168/205) gave birth during the study period, most frequently within 48 hours of presentation (see Appendix B, available from: http://emed.stanford.edu/education/international/pubs.html). In addition, 28% (65/232) of women with pregnancy-related complaints underwent obstetric surgery. No maternal deaths were noted during follow-up.

Multivariate logistic regression analysis identified several factors associated with an increased risk of admission (*n* = 729, *c*-statistic: 0.815). Patients who presented with an acute or recent complaint were more likely to be admitted than those who did not (OR: 5.09; 95% CI: 3.49–7.42). Similarly, the likelihood of admission was increased in those with abnormal blood pressure, respiratory rate or heart rate (OR: 3.22; 95% CI: 2.09–4.95) or fever (OR: 3.01; 95% CI: 1.50–6.03). Multivariate analysis also identified factors associated with an increased risk of surgery (*n* = 450, *c*-statistic: 0.776). Surgery was more likely in patients with a genitourinary complaint (OR: 3.46; 95% CI: 1.78–6.75) or a traumatic injury (OR: 2.33; 95% CI: 1.14–4.78) and in those who presented at Battambang Provincial Hospital (OR: 3.83; 95% CI: 2.17–6.78). Conversely, surgery was less likely in patients with an abnormal blood pressure, respiratory rate or heart rate (OR: 0.51; 95% CI: 0.28–0.91). Predictors of functional impairment at 14 days identified by multivariate analysis (*n* = 397, *c*-statistic: 0.668) were traumatic injury (OR: 2.16; 95% CI: 1.12–4.16) or an acute complaint (OR: 0.55; 95% CI: 0.33–0.92). Details of the multivariate analyses are presented in Appendices C, D and E (available from: http://emed.stanford.edu/education/international/pubs.html).

## Discussion

This study of adults presenting without an appointment at two large public referral hospitals in Cambodia helps to describe the patient population seeking acute care in Cambodia and South-East Asia. Demographic data showed that the patients’ mean age was 18 years older than the median age of the Cambodian population. In addition, 12.6% of the study cohort was aged 65 years or more, whereas only 7.7% of the country’s population is older than 60 years.^11^

The most common chief complaint was abdominal pain, which accounted for over one third of visits and 45% of admissions. Other gastrointestinal complaints, such as vomiting and diarrhoea, were infrequent. The next most common chief complaints were respiratory problems, headache, injury and chest pain, respectively. Few comparative studies of chief complaints at emergency departments in health-care systems in low- and middle-income countries have been reported in the literature. A recent study at a single health centre in Kenya found that road traffic injuries, vaginal bleeding and altered mental status were the most common chief complaints; the most common emergency department diagnosis was injury (20%).^12^ In our study, injuries accounted for 11.3% of visits and one death. Fever ranked seventh as the primary reason for a hospital visit in our study and was the ninth most common reason in the Kenyan study.^12^ Although infectious disease still accounts for a large proportion of the disease burden in low- and middle-income countries, our results are consistent with previous research which suggests that noncommunicable disease is an increasingly common reason for seeking acute care.^5^^,^^13^^,^^14^ Abdominal pain is reportedly the chief complaint of 9% of adults seeking care at emergency departments in the United States of America.^15^ According to these nationally-representative statistics, 7% of adults presented with chest pain while headache, shortness of breath and back pain each accounted for 3% of emergency department visits.

Our observations in Cambodia show that diagnostic tests are seldom used during the first 48 hours following an emergency admission. Electrocardiography, radiography and ultrasound imaging were available at the two hospitals. This meant that standard evaluations were not done for most chief complaints. The lack of diagnostic evaluations was probably due to both limited resources and inadequate training. We found that over 40% (572) of patients held government-subsidized health equity fund insurance cards. Since the health equity fund awards a fixed amount per patient-visit and many employed people live in poverty, patients may not have been able to pay for even low-cost diagnostic tests.

Poor diagnostic and treatment practices may be due in part to gaps in continuing medical education. Currently, continuing education is not mandatory for health-care providers in Cambodia. Furthermore, most educational programmes approved by the Ministry of Health concentrate on identifying and managing infectious diseases, such as tuberculosis, and on maternal care. Although it is important to address common infectious diseases, our data suggest that the majority of patients present with undifferentiated complaints caused by noncommunicable diseases. Focusing diagnostic resources and provider education on the most common conditions would improve the evaluation and management of these patients.

This study identified several simple, yet important, changes that could potentially improve health-care delivery at Cambodian referral hospitals. First, despite a low number of deaths in the short term, a high percentage of patients were admitted (60.5%). Comparable studies in other low- and middle-income countries, report about 40% admissions.^16^ Identifying patients who can be safely discharged would substantially reduce unnecessary admissions and, therefore, health-care costs. Most referral hospitals in Cambodia do not have a central point of entry for patients seeking treatment with undifferentiated complaints. Moreover, neither hospital in our study had a triage system. In the absence of an emergency department and a triage system, inefficiencies, delays in care and inconsistencies in treatment are likely to arise. Consequently, admission criteria at the two study hospitals may have varied according to where in the hospital the patient presented. Relatively simple changes could address these deficits, such as deploying a triage system, designating an area for evaluating new patients and improving staff capacity for managing patients with undifferentiated complaints.

Second, nearly half of the study patients had previously consulted an outside provider for their presenting complaint. There is no coordinated system in Cambodia for referring patients, for sharing medical information between facilities or for transferring patients.^17^ Strengthening health management information systems and introducing phone-based referral centres could improve communications between government facilities, save time and help avoid repeating procedures during the evaluation and treatment of patients.

Finally, vital signs were not reliably measured or reported at either study hospital. For approximately one in five patients, temperature was not measured. A similar proportion of patients had no records of respiratory rate, blood pressure and heart rate. Again, the absence of a central point of entry and of a triage system for new patients may have contributed to inconsistencies in measurements or documentation. As vital signs are important markers of the severity of illness and have been shown to be reliable predictors of death following trauma and acute poisoning in low- and middle-income countries, these should be measured and documented in every patient.^18^^,^^19^

The study had several limitations: patients were not enrolled 24 hours a day, seven days a week; reliable discharge diagnoses could not always be obtained; and it was difficult to track discharged patients. Although our findings may not be generalizable to other locations, the two study sites did serve different communities and provinces.

In summary, we have characterized the chief complaints, initial care and outcomes of adults seeking emergency care in two public hospitals in Cambodia. We identified several opportunities for operational and educational improvements, including better documentation of vital signs and increased use of diagnostic techniques. Further study is required to elucidate the factors underlying the high admission-to-death ratio and the limited use of diagnostic tests.
